# Myeloid tissue factor does not modulate lung inflammation or permeability during experimental acute lung injury

**DOI:** 10.1038/srep22249

**Published:** 2016-02-29

**Authors:** Ciara M. Shaver, Brandon S. Grove, Jennifer K. Clune, Nigel Mackman, Lorraine B. Ware, Julie A. Bastarache

**Affiliations:** 1 Division of Allergy, Pulmonary, and Critical Care Medicine, Department of Medicine, Vanderbilt University Medical Center, Nashville, TN, USA; 2 Division of Hematology and Oncology, UNC McAllister Heart Institute, University of North Carolina at Chapel Hill, Chapel Hill, NC, USA; 3 Department of Pathology, Microbiology, and Immunology, Vanderbilt University Medical Center, Nashville, TN, USA.

## Abstract

Tissue factor (TF) is a critical mediator of direct acute lung injury (ALI) with global TF deficiency resulting in increased airspace inflammation, alveolar-capillary permeability, and alveolar hemorrhage after intra-tracheal lipopolysaccharide (LPS). In the lung, TF is expressed diffusely on the lung epithelium and intensely on cells of the myeloid lineage. We recently reported that TF on the lung epithelium, but not on myeloid cells, was the major source of TF during intra-tracheal LPS-induced ALI. Because of a growing body of literature demonstrating important pathophysiologic differences between ALI caused by different etiologies, we hypothesized that TF on myeloid cells may have distinct contributions to airspace inflammation and permeability between direct and indirect causes of ALI. To test this, we compared mice lacking TF on myeloid cells (TF^∆mye^, LysM.Cre^+/−^TF^flox/flox^) to littermate controls during direct (bacterial pneumonia, ventilator-induced ALI, bleomycin-induced ALI) and indirect ALI (systemic LPS, cecal ligation and puncture). ALI was quantified by weight loss, bronchoalveolar lavage (BAL) inflammatory cell number, cytokine concentration, protein concentration, and BAL procoagulant activity. There was no significant contribution of TF on myeloid cells in multiple models of experimental ALI, leading to the conclusion that TF in myeloid cells is not a major contributor to experimental ALI.

The acute respiratory distress syndrome (ARDS), a severe form of acute lung injury (ALI), causes more than 75,000 deaths annually in the United States^1^ and has no specific therapies aside from use of low tidal volume ventilation and conservative fluid management^2^,^3^. Most commonly, ARDS occurs in the setting of direct lung injury from bacterial pneumonia or in the setting of indirect lung injury from non-pulmonary sepsis^4^,^5^. The mechanisms underlying development of ALI and ARDS remain unclear.

Tissue factor (TF) is a trans-membrane protein critical for activation of the coagulation cascade during hemostasis as well as in response to tissue injury. In the lung, TF is expressed primarily on the lung epithelium and on cells of the myeloid lineage (monocytes, macrophages, and neutrophils)^6^,^7^,^8^,^9^,^10^. In a model of direct ALI caused by intra-tracheal delivery of lipopolysaccharide (LPS), global reduction of TF resulted in increased airspace inflammation and increased alveolar-capillary barrier permeability^11^, suggesting that TF is protective during direct ALI. In contrast, other studies using animal models of indirect ALI have suggested that TF deficiency or systemic TF inhibition reduced tissue injury^12^,^13^,^14^,^15^. We recently identified that TF expressed on the lung epithelium was the predominant source of TF in the lung during direct LPS-induced ALI^16^, with no significant contribution of TF from myeloid cells in this model. This finding was in contrast to prior data showing that TF expressed on cells in the circulation was critical for the systemic response to LPS in a model of endotoxemia^17^. Because of these apparent differences in the role of TF in direct ALI compared to indirect ALI and because of the critical role of macrophages and neutrophils in pulmonary host defense, we sought to better understand the role of myeloid TF in different models of ALI. Using mice with a targeted genetic deletion of TF only in cells of the myeloid lineage, we tested the hypothesis that TF on myeloid cells would be protective in models of direct ALI but detrimental in models of indirect ALI.

## Results

### Myeloid TF in direct lung injury

To test the impact of myeloid cell TF in acute lung injury, mice with myeloid cell TF deletion (LysM.Cre^+/−^TF^flox/flox^, referred to as TF^∆mye^) and littermate controls (LysM.Cre^−/−^TF^flox/flox^, wild-type, WT) were subjected to multiple models of ALI. Circulating blood cells from TF^∆mye^ mice have been previously shown to have abrogated expression of TF mRNA during endotoxemia and peripheral blood cells from TF^∆mye^ mice had a 93% reduction in pro-coagulant activity after *in vitro* LPS stimulation^17^. We independently confirmed deletion of myeloid TF in our colony of TF^∆mye^ mice. Alveolar macrophages isolated from TF^∆mye^ mice that were stimulated *ex vivo* with LPS had TF mRNA levels that were 90% lower than in cells isolated from wild-type littermates (p = 0.019). Similarly, thioglycollate-induced peritoneal macrophages isolated from TF^∆mye^ mice had TF mRNA levels 89.5% lower after LPS stimulation than cells isolated from wild-type littermates (p = 0.019). These studies confirm that myeloid cells in our mouse model have a functional defect in TF expression and activity.

**Figure 1:**
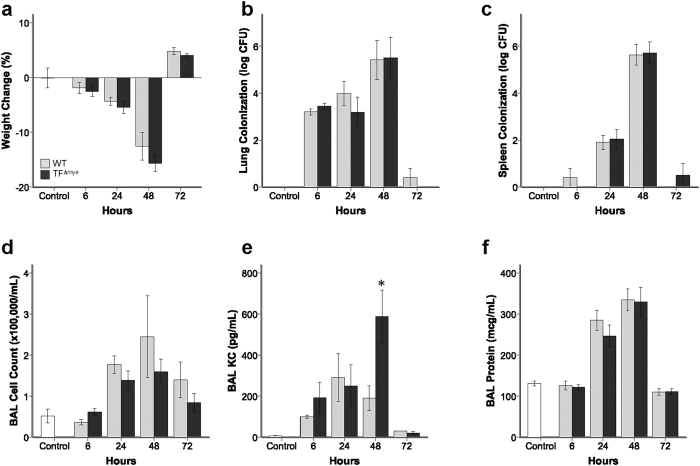
Effect of myeloid tissue factor deletion during acute *Klebsiella pneumoniae* infection. There were no significant differences between wild-type mice (gray bars) and mice lacking myeloid tissue factor (TF^∆mye^) (black bars) in (**a**) weight change, (**b**) bacterial colonization in the lung, (**c**) bacterial colonization in the spleen, and (**d**) total inflammatory cell count in bronchoalveolar lavage (BAL) fluid. There was a statistical difference in (**e**) BAL KC/CXCL-1 between genotypes only at the 48hr time point (*p = 0.023). There were no significant differences between WT and TF^∆mye^ mice in (**f**) BAL total protein. Analysis includes 4–21 mice in each genotype at each time point. Statistical comparisons were performed with Student’s *t* tests comparing genotypes at each time point. Data from uninfected controls (white bars) are included for reference.

**Figure 2:**
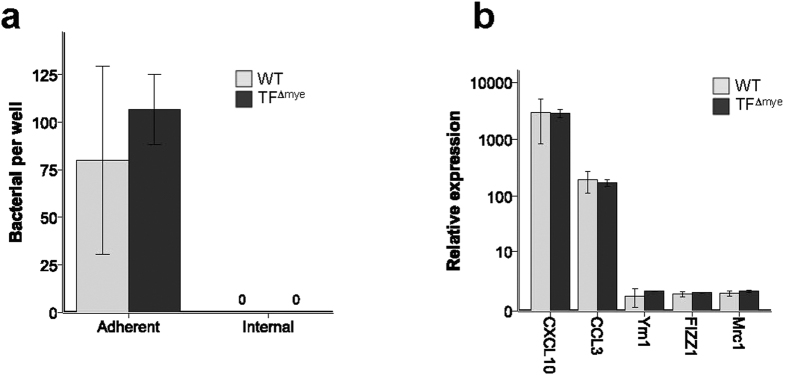
Effect of deletion of myeloid TF on macrophage phagocytosis and cytokine production. (**a**) Thioglycollate-induced peritoneal macrophages from mice lacking myeloid TF (TF^∆mye^) and littermate controls have similar attachment, phagocytosis, and killing of *Klebsiella pneumoniae* at multiplicity of infection (MOI) of 10. Similar results were obtained at MOI of 100, 500, and 1000. (**b**) Thioglycollate-induced peritoneal macrophages from TF^∆mye^ mice and littermate controls had increased expression of M1 macrophage markers (CXCL10, CCL3) with no upregulation of M2 markers (Ym1, FIZZ1, Mrc1) in response to *ex vivo* LPS stimulation, with no difference between WT cells and those lacking TF (results normalized to PBS-treated controls).

We previously demonstrated that TF on myeloid cells had no significant impact on ALI due to IT LPS administration^16^. However, because of the critical role of myeloid cells in coordination of pulmonary innate immunity in response to bacterial infection, we hypothesized that TF on myeloid cells may have a prominent role during bacterial pneumonia. To test this possibility, mice were infected with *Klebsiella pneumoniae*, a common cause of bacterial pneumonia. WT mice developed significant weight loss (Fig. 1a) associated with increased bacterial colonization in the lung and spleen (Fig. 1b,c), which resolved after 72 hours. BAL inflammatory cells (Fig. 1d), KC/CXCL-1 expression (Fig. 1e), and total BAL protein (Fig. 1f) peaked after 48 hrs in WT mice. Deletion of myeloid TF had no impact in this model system. WT and TF^∆mye^ animals had indistinguishable bacterial colonization in the lung and similar bacterial dissemination to the spleen. Mice lacking myeloid TF had similar neutrophilic BAL inflammation throughout the experiment (data not shown). Expression of KC in BAL of TF^∆mye^ mice was variable over time, with mice lacking myeloid TF having more BAL KC only at the 48 hr assessment, a finding of uncertain biological significance (Fig. 1e). In addition, isolated peritoneal macrophages from WT and TF^∆mye^ mice had similar phagocytosis and bacterial killing of *K. pneumoniae in vitro* (Fig. 2a). Furthermore, peritoneal macrophages isolated from WT and TF^∆mye^ mice induce pro-inflammatory M1 macrophage markers, but not M2 macrophage markers, in response to LPS *in vitro* (Fig. 2b). These data confirm that lack of TF had no effect on antimicrobial macrophage functions.

**Figure 3:**
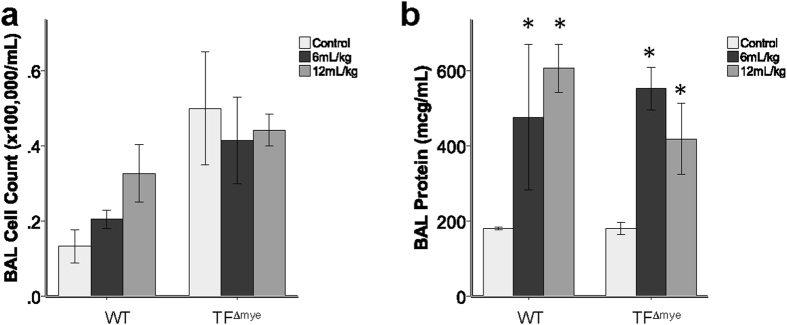
Effect of myeloid tissue factor deletion during mechanical ventilation. There were no significant differences between wild-type (WT) mice and mice lacking myeloid tissue factor (TF^∆mye^) in (**a**) total inflammatory cell counts in bronchoalveolar lavage (BAL) fluid or (**b**) BAL total protein after 2 hours of mechanical ventilation at either low (6 mL/kg) or high (12 mL/kg) tidal volumes. *indicates p < 0.05 compared to control treated animals in each genotype. The differences between ventilated and non-ventilated control mice are significant showing that this model induces lung injury in WT and TF^∆mye^ mice. Analysis includes 2–5 mice in each genotype. Statistical comparisons were performed by two-way ANOVA.

Next, we tested whether mice lacking TF on myeloid cells had altered susceptibility to mechanical lung injury caused by mechanical ventilation. As expected, mechanical ventilation with both low (6 mL/kg) and high (12 mL/kg) tidal volumes induced a significant increase in BAL protein with limited BAL inflammation in WT mice. There was no significant impact of myeloid TF on BAL inflammation (Fig. 3a) or BAL protein (Fig. 3b) in this model of direct lung injury. BAL levels of cell-free hemoglobin were below the limit of detection (10 mg/dL) in both WT and TF^∆mye^ animals (data not shown).

**Figure 4:**
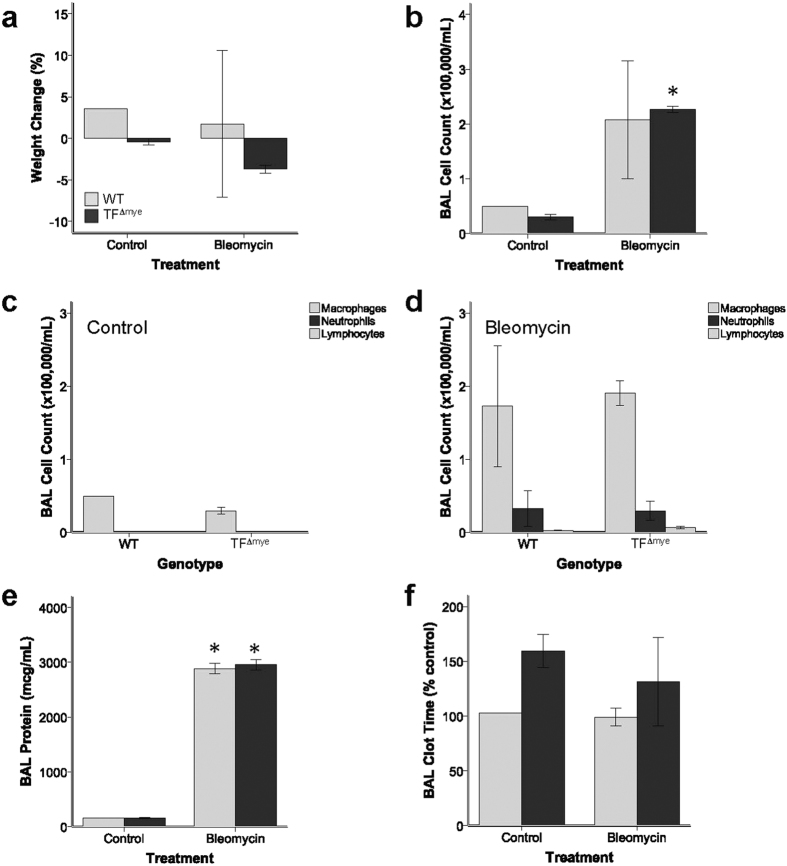
Effect of myeloid tissue factor deletion after intra-tracheal bleomycin administration. There were no significant differences between wild-type (WT) mice and mice lacking myeloid tissue factor (TF^∆mye^) in (**a**) weight change, (**b**) total inflammatory cell counts in bronchoalveolar lavage (BAL) fluid, (**c,d**) BAL differential cell counts, (**e**) BAL total protein, or (**f**) BAL clot time at 7 days after bleomycin or control. *indicates p < 0.05 compared to control treated animals in each genotype. Analysis includes 2–3 mice in each genotype. Statistical comparisons were performed with Student’s *t* tests.

We also tested whether myeloid TF had a more prominent role during prolonged inflammation using a model of lung injury associated with fibrosis. Mice were treated with intra-tracheal bleomycin and samples collected after 7 days, which coincides with the peak of lung inflammation in this model. WT and TF^∆mye^ mice had similar weight changes (Fig. 4a), BAL inflammatory cell counts (Fig. 4b), BAL cell differentials (Fig. 4c,d), BAL protein (Fig. 4e), and BAL clot times (Fig. 4f). BAL levels of cell-free hemoglobin were below the limit of detection (10 mg/dL) in both WT and TF^∆mye^ animals (data not shown).

### Myeloid TF during indirect acute lung injury

**Figure 5:**
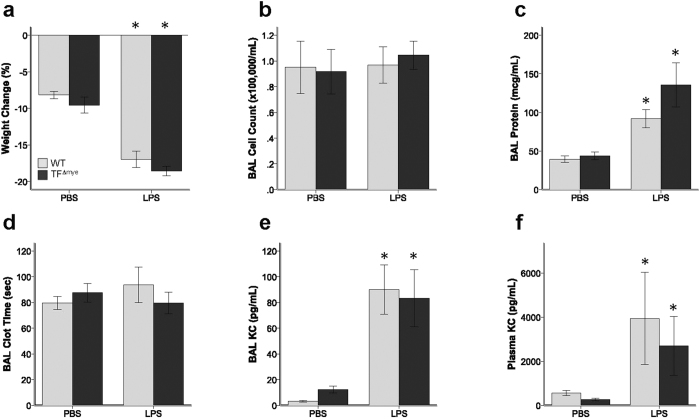
Effect of myeloid tissue factor deletion during systemic lipopolysaccharide administration. Mice were treated with continuous LPS infusion (8 μg/hr) via implanted osmotic pump and samples collected after 48 hours. There were no significant differences between wild-type (gray bars) mice and mice lacking myeloid tissue factor (black bars) in (**a**) weight change, (**b**) total inflammatory cell count in bronchoalveolar lavage (BAL) fluid, (**c**) BAL total protein, (**d**) BAL clot time, (**e**) BAL KC/CXCL-1, or (**f**) plasma KC. *indicates p < 0.05 compared to control treated animals in each genotype. Analysis includes 4–21 mice in each genotype at each time point. Statistical comparisons were performed by Student’s *t* test.

Indirect ALI during sepsis is predominantly an endothelial injury, with secondary acute lung injury in part due to vascular leak into the alveolar space^5^. We hypothesized that myeloid TF may have a more prominent role during this injury because of activation of circulating inflammatory cells during sepsis. Systemic delivery of LPS over 48 hours in WT mice resulted in significant increases in weight loss (Fig. 5a), BAL protein (Fig. 5c), BAL KC (Fig. 5e), and plasma KC (Fig. 5f) as compared to PBS-treated controls, demonstrating that this model causes significant systemic inflammation, increased lung permeability, and elevated airspace chemokines. Contrary to our hypothesis, there were no differences between WT and TF^∆mye^ mice in weight loss (Fig. 5a), BAL cell counts (Fig. 5b), BAL protein (Fig. 5c), BAL KC (Fig. 5e), or plasma KC (Fig. 5f) levels. There was no significant activation of airspace coagulation (Fig. 5d) in this model. BAL levels of cell-free hemoglobin were below the limit of detection (10 mg/dL) in both WT and TF^∆mye^ animals (data not shown).

**Figure 6:**
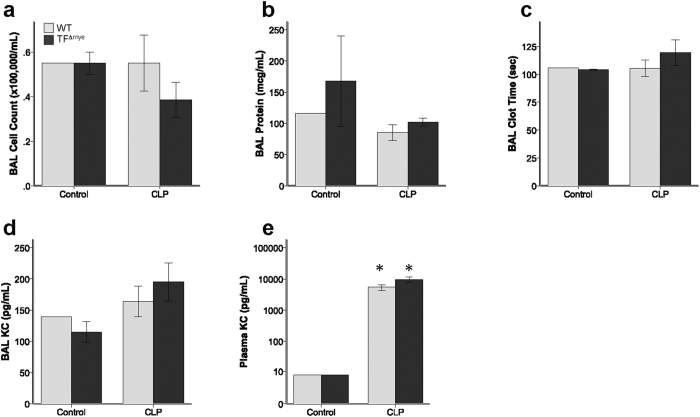
Effect of myeloid tissue factor deletion after cecal ligation and puncture. Wild-type (WT) mice and mice lacking myeloid tissue factor (TF^∆mye^) had similar (**a**) total inflammatory cell counts in bronchoalveolar lavage (BAL) fluid, (**b**) BAL total protein, (**c**) BAL clot time, (**d**) BAL KC/CXCL-1, and (**e**) plasma KC. *indicates p < 0.05 compared to control treated animals in each genotype. Analysis includes 1–2 sham mice and 11–14 CLP mice in each genotype. Statistical comparisons were performed by Student’s *t* tests.

Finally, we tested whether myeloid TF had a significant role in the response to polymicrobial sepsis caused by cecal ligation and puncture (CLP), a common model of sepsis-associated end-organ injury. Although CLP does not cause a robust acute lung injury^18^, we hypothesized that loss of myeloid TF would exacerbate lung injury in this model. Both WT and TF^∆mye^ mice had limited BAL inflammation (Fig. 6a), BAL protein (Fig. 6b), BAL procoagulant activity (Fig. 6c), and BAL KC expression (Fig. 6d), with no differences between groups. Both groups of mice had increased plasma KC levels compared to sham treated mice (Fig. 6e) with no difference between genotypes in this measure of systemic inflammation. BAL levels of cell-free hemoglobin were below the limit of detection (10 mg/dL) in both WT and TF^∆mye^ animals (data not shown).

## Discussion

In this study, we demonstrate that deletion of TF on cells of the myeloid lineage has no detectable impact during acute lung injury caused by multiple models of direct or indirect lung injury. While these findings are consistent with our previous report of a limited effect of myeloid TF during intra-tracheal LPS-induced acute lung injury, the lack of any effect of myeloid TF is very interesting given the robust upregulation of TF in myeloid cells in models of lung injury. Several studies have shown a major role for TF in mediating coagulant and inflammatory responses in sepsis^8^,^12^,^13^,^14^,^19^,^20^,^21^. TF deficiency or inhibition reduced indirect acute lung injury and improved mortality in experimental models of sepsis^14^,^22^,^23^. Baboons with experimental sepsis treated with site-activated factor VII (which prevents TF-induced coagulation) developed less ALI^14^. In a similar study, baboons given TF-pathway-inhibitor (TFPI) in the setting of bacteremia had reduced mortality^22^, suggesting a key role for TF in outcomes of sepsis. However, it should be pointed out that TFPI administration likely has additional effects that differ from genetic deletion of TF so it is difficult to make a direct comparison. In addition, after a single injection of LPS, mice with relative TF deficiency had less cytokine induction and increased survival^23^. These preclinical data were so compelling that two clinical trials of systemic TF inhibition in human sepsis and in community-acquired pneumonia were performed, each failing to show clinical benefit with one study showing higher mortality with TF inhibition during pneumonia^24^,^25^. Although these studies suggest that TF is pro-inflammatory and pro-injurious, none of them studied cell-specific effects of TF. Our study of myeloid TF in multiple different direct and indirect ALI models convincingly establishes that myeloid TF does not play a significant role in ALI pathogenesis.

Another distinction is that many of these studies focused on the impact of TF inhibition on systemic illness rather than addressing the role of TF specifically in the injured lung. TF activation is necessary for fibrin deposition, which may attenuate alveolar permeability. Pawlinski *et al.* showed that TF on circulating hematopoietic cells was sufficient for activation of coagulation in plasma after exposure to systemic LPS^17^. In contrast, our group has previously shown that mice that are globally-deficient in TF (low TF mice) have increased airspace inflammation and increased lung permeability after direct LPS-mediated ALI^11^. This result prompted our additional investigation of the role of myeloid TF during lung injury. However, our current studies demonstrate no major effect of myeloid TF in multiple models of either direct or indirect lung injury, even when the injury is non-infectious. Furthermore, the lack of difference in systemic inflammation during polymicrobial sepsis between WT and myeloid TF-deficient animals is surprising and is contrary to our hypothesis that loss of myeloid TF would cause lung inflammation in these models that do not typically result in significant lung inflammation. Our results suggest that other cellular sources of TF are the primary mediators of coagulation and inflammation in response to tissue injury. Consistent with this concept, loss of TF on the lung epithelium exacerbates direct ALI^16^, whereas TF on the vascular endothelium may be most critical in models of sepsis as suggested by Pawlinski *et al.*^17^.

In the lung, TF is expressed on cells of the myeloid lineage, including monocytes, macrophages, and neutrophils^6^,^8^,^11^,^16^,^26^,^27^, albeit at total levels less than seen on the lung epithelium. Lung biopsies of patients with ARDS show strong staining for TF in alveolar macrophages^6^. *In vitro*, macrophages robustly upregulate TF expression after LPS stimulation. Similarly, neutrophils have been shown to immunostain for TF^28^,^29^. Thus, we were surprised that myeloid TF had no demonstrable effect during ALI. We add to the known literature of TF in macrophages by demonstrating that genetic deletion of TF from myeloid cells had no significant impact on macrophage phagocytosis or microbicidal functions (Fig. 2a) and no effect on cytokine production or macrophage polarization in response to LPS (Fig. 2b).

There are several potential explanations for our findings. The simplest explanation is that TF on other cell types contributes the majority of TF in the lungs. We recently showed that the lung epithelium contributes more than 70% of total lung TF both basally and in response to LPS^16^. Despite alveolar macrophages from TF^∆mye^ mice having only 10% of WT TF mRNA levels, the overall amount of TF protein expressed in the injured lung of TF^∆mye^ mice is indistinguishable from WT animals^16^. While macrophages often have intense TF staining on an individual cell, the majority of total TF in the lung is expressed on the lung epithelium. Another explanation for the lack of impact of myeloid TF in these models of ALI is that the importance of myeloid TF is obscured in the presence of TF on other cells such as the lung epithelium. In addition, while TF on the endothelium had no detected role in activation of coagulation in plasma after systemic LPS administration^17^, there are few data on the impact of TF on endothelial cells in other systems. Finally, because the model systems used cause relatively mild inflammatory lung injury, a role for myeloid TF in inflammation and coagulation during severe lung injury remains possible.

In conclusion, despite extensive data that supports a role for TF in acute lung injury and sepsis and the substantial body of literature showing strong induction of TF expression in myeloid cells, we were unable to demonstrate any biologically significant role for TF expressed on myeloid cells in multiple models of both direct and indirect acute lung injury. In the lung, further investigation of the role of TF during ALI should focus on its protective role on the lung epithelium and its effects on maintenance of alveolar-capillary barrier integrity during direct ALI.

## Material and Methods

### Transgenic mice

Mice with myeloid cell TF deletion (LysM.Cre^+/−^TF^flox/flox^, TF^∆mye^) and littermate controls (LysM.Cre^−/−^TF^flox/flox^, wild-type, WT) on a C57Bl/6 background were used for all experiments. Generation of TF^∆mye^ animals has been previously described^16^,^17^. Male and female mice aged 7–18 weeks were used for these studies. All experiments were approved by the Vanderbilt Institutional Animal Care and Use Committee and performed in accordance with approved procedures (protocol M12–221).

### Quantification of tissue factor expression

Alveolar macrophages were collected by bronchoalveolar lavage of untreated wild type and TF^∆mye^ mice, adhered to tissue culture dishes for 2 hours, and washed. Peritoneal macrophages were collected from the abdomen in 10 mL cold PBS 3 days after intraperitoneal 3% thioglycollate administration. Alveolar and peritoneal macrophages were washed and then stimulated *in vitro* with 10 μg/mL LPS for 2 hours. RNA was collected with a PureLink RNA extraction kit (Ambion, Grand Island, NY) and cDNA generated using SuperScript VILO reagents (Invitrogen, Life Technologies, Grand Island, NY). TF mRNA expression was measured by quantitative real time PCR using primers for murine TF according to established methods^11^.

### Measurements of acute lung injury

At each time point in each model of ALI, mice were euthanized and plasma was collected in citrate. Bronchoalveolar lavage (BAL) was performed with 900 μl PBS as previously described^11^,^16^. Lungs were removed and flash frozen. All samples were stored at −80 °C until further study. For bacterial pneumonia experiments, one lung was separated and homogenized in PBS for bacterial quantification prior to BAL of the remaining lung with 600 μl PBS. BAL clot time, cell counts, protein, and cytokine concentrations were measured as previously described^11^,^16^,^30^,^31^.

### Bacterial pneumonia

*Klebsiella pneumoniae* serotype 2 (ATCC strain #43816, courtesy of R. Stokes Peebles, Vanderbilt University, Nashville, TN) was grown overnight in Luria Bertani medium, diluted in fresh medium, and grown for 1–2 hours prior to preparation of the inoculum. Mice were anesthetized with ketamine and xylazine and 2 × 10^3^ colony forming units were administered intra-nasally^32^. Samples were collected after 6, 24, 48, or 72 hours.

### Ventilator-induced lung injury

Mice were anesthetized with repeated injections of pentobarbital (5 mg/g mouse). After undergoing tracheotomy, mice were either mechanically ventilated with either low (6 mL/kg) or high (12 mL/kg) tidal volumes, respiratory rate 40 breaths/min per L tidal volume, and positive end-expiratory pressure of 2 cm H_2_O or monitored without ventilation for 2 hours prior to sample collection.

### Bleomycin-induced lung injury

Mice were anesthetized with isoflurane and 0.04 U bleomycin sulfate (Bristol-Myers Squibb Co., Princeton, NJ) in 100 μl PBS was administered IT as previously described^11^. Samples were collected after 7 days.

### Systemic LPS-induced lung injury

Indirect LPS-induced lung injury was induced by continuous infusion of LPS (8 μg/hr for 24 hrs) (*Escherichia coli*, LPS, Sigma, St. Louis, MO) or equal volume PBS delivered by intra-peritoneal osmotic pump (Durect, Cupertino, CA) with a single intra-peritoneal dose of LPS (3 μg/mouse) or PBS at the time of pump implantation^16^,^33^. Samples were collected after 48 hours.

### Cecal ligation and puncture

To induce indirect lung injury by cecal ligation and puncture (CLP), mice were sedated with isoflurane and a midline laparotomy performed. The cecum was identified, ligated with suture, and punctured once with a 23-gauge needle. The peritoneum and skin were closed with sutures. Mice were frequently monitored for discomfort and given narcotic pain medication preoperatively and as needed postoperatively. Samples were collected after 24 hours.

### Macrophage phagocytosis and cytokine expression

Thioglycollate-elicited peritoneal macrophages were collected as described above and incubated with *K. pneumoniae in vitro* at a multiplicity of infection of 10. After 2 hours, cells were washed x 3 with PBS. Adherent bacteria were quantified by plating of cell lysates on LB agar. Internalized bacteria were quantified after treatment of triplicate wells with gentamicin for 1 hour prior to being washed x 3 with PBS. For cytokine expression, RNA was collected from peritoneal macrophages after 3 hours of incubation with LPS. Data for LPS-treated cells collected from individual mice are normalized to PBS-treated macrophages from the same mouse.

### Statistical Analysis

All statistical analyses were done with SPSS version 22 for Macintosh with statistical consultation. P < 0.05 was considered statistically significant. Wild type and TF^∆mye^ were compared in each treatment group by Student’s *t* tests. Non-normally distributed data were natural log transformed prior to analysis.

## Additional Information

**How to cite this article**: Shaver, C. M. *et al.* Myeloid tissue factor does not modulate lung inflammation or permeability during experimental acute lung injury. *Sci. Rep.* **6**, 22249; doi: 10.1038/srep22249 (2016).
